# Lipid Remodeling in Mouse SR-B1-Deficient Embryos with Oxidative Stress-Associated Neural Tube Defects

**DOI:** 10.3390/antiox15050634

**Published:** 2026-05-16

**Authors:** Alonso Quiroz, Nicolás Santander, Greene D. E. Nicolás, Kit-Yi Leung, Dolores Busso

**Affiliations:** 1 Ph.D. Program in Medical Science, Faculty of Medicine, Pontificia Universidad Católica de Chile, Santiago 8331150, Chile; 2 Institute of Health Sciences, Universidad de O’Higgins, Rancagua 2820000, Chile; 3 Great Ormond Street Institute of Child Health, University College London, London WC1N 1EH, UK; 4 Biomedical Research and Innovation Center, Faculty of Medicine, Universidad de Los Andes, Santiago 7550000, Chile; 5 Center of Interventional Medicine for Precision and Advanced Cellular Therapy (IMPACT), Santiago 7550000, Chile

**Keywords:** neural tube defects, oxidative stress, SR-B1, lipidomics, transcriptomics

## Abstract

Neural tube defects (NTD) are congenital malformations that lead to structural abnormalities of the brain or spine. Mouse embryos deficient in Scavenger Receptor Class B Type 1 (SR-B1 KO), the main receptor for high-density lipoproteins, exhibit a high incidence of anterior NTD, which is associated with vitamin E deficiency and elevated levels of reactive oxygen species (ROS). Maternal supplementation with vitamin E, a micronutrient with antioxidant properties, completely prevents the occurrence of NTD and normalizes ROS levels in SR-B1 KO embryos, suggesting a contribution of oxidative stress to NTD in this model. In this work, we showed that SR-B1 KO embryos at gestational day E9.5 display higher levels of lipoperoxidative damage markers. Analysis of data obtained through shotgun lipidomics evidenced a selective and coordinated reorganization of fatty acid distribution, characterized by altered polyunsaturated and monounsaturated composition, together with reduced phosphatidylcholine and increased lysophosphatidylcholine levels, and diversion of fatty acids into triacylglyceride storage. Transcriptomic analysis revealed a coordinated upregulation of genes involved in phospholipid synthesis and remodeling, consistent with the altered lipid homeostasis observed in SR-B1 KO embryos. Together, these results provide novel information showing a potential link between oxidative stress and disruptions in mammalian embryonic lipid metabolism, highlighting phospholipid remodeling as a potential determinant of susceptibility to NTD.

## 1. Introduction

Scavenger receptor class B type 1 (SR-B1) is a high-affinity receptor for high-density lipoproteins that facilitates the selective uptake of cholesteryl esters (CEs) and lipid-soluble micronutrients, including vitamin E [1,2,3]. This vitamin is one of the major lipid-phase antioxidants in biological membranes [4]. SR-B1 is widely expressed in tissues with high metabolic or endocrine activity, such as the liver, adrenal glands, and ovaries, where it plays a critical role in lipid transport and homeostasis [5]. Consistent with the role of this receptor, SR-B1 knock-out (KO) adult mice show vitamin E deficiency in several tissues, including the brain, lungs, and gonads [6].

Mice are considered a valuable experimental model to study human malformations arising from intrauterine development. Our laboratory previously reported that SR-B1 KO mouse embryos exhibit a high penetrance of exencephaly or cranial neural tube defect (NTD) [7,8,9]. In the 129:C57Bl/6J background, the incidence of the malformation is around 1:2 (50%), with a female skew [7]. Despite this early malformation of the nervous system observed in SR-B1 KO embryos, SR-B1 protein is not expressed in the embryo itself during neural tube closure but is localized in trophoblast giant cells forming the parietal yolk sac (pYS), a structure responsible for the early steps of maternal–fetal nutrient transport [7,10]. Given that SR-B1 is expressed in the pYS rather than in the embryo proper, molecular changes in SR-B1 KO embryos likely arise from a coordinated interplay between extraembryonic lipid uptake and embryonic metabolic responses. Indeed, SR-B1 KO embryos show severe vitamin E deficiency and increased levels of reactive oxygen species (ROS) compared to their wild-type (WT) littermates [11]. In previous studies, we showed that feeding dams a vitamin E-enriched diet completely rescued the NTD phenotype and reduced ROS levels in SR-B1 KO embryos [8,9,11], supporting a contribution of oxidative stress to NTD development in this model.

Evidence from human and rodent studies supports oxidative stress as a major etiological factor in NTD [12]. Increased markers of lipoperoxidation, together with reduced antioxidant defenses, have been detected in maternal serum and amniotic fluid from NTD-affected pregnancies [13,14,15]. Maternal diabetes represents a well-characterized example of oxidative stress-linked NTD, as hyperglycemia enhances embryonic mitochondrial ROS production and disrupts redox-sensitive pathways that regulate proliferation, apoptosis, and neural differentiation [16,17,18,19]. Other environmental teratogens, including benzo[a]pyrene [20], arsenic [21], ethanol [22], valproic acid [23], and retinoic acid [24], similarly increase NTD risk through oxidative stress-mediated mechanisms. Importantly, antioxidant interventions reduce NTD incidence in several experimental models, further supporting a pathogenic role for oxidative stress during neural tube closure [18,19,23,25].

Membrane lipids are among the cellular components most vulnerable to oxidative stress, and phospholipids are particularly susceptible because they contain polyunsaturated fatty acids (PUFAs), which readily undergo oxidation due to the presence of conjugated systems. Reactive oxygen species-mediated oxidation of PUFA-containing phospholipids alters membrane biophysical properties and generates reactive lipid peroxidation products capable of damaging proteins, nucleic acids, and other biomolecules [26]. In addition, phospholipid remodeling through phospholipase-mediated hydrolysis can increase lysophospholipids [27]. Because PUFAs also serve as precursors of bioactive lipid mediators involved in embryogenesis, alterations in membrane lipid composition may have important developmental consequences [28,29,30]. Vitamin E and PUFA metabolism are closely interconnected. Vitamin E interrupts lipid oxidation reactions by donating a hydrogen atom to lipid peroxyl radicals, thereby protecting PUFAs from oxidative damage, and also modulates gene expression, signal transduction pathways, and enzymatic processes involved in inflammation, lipid metabolism, and cell proliferation [31]. In humans, vitamin E requirements increase in parallel with dietary PUFA intake and their degree of unsaturation [32]. Similarly, in pregnant rats fed a fish oil-enriched diet, which is rich in highly unsaturated fatty acids, vitamin E concentrations are reduced in both maternal plasma and fetal tissues compared with animals fed an olive oil-based diet [33].

The developmental consequences of oxidative stress on lipids have been most extensively studied in zebrafish. When fed a vitamin E-deficient diet, these animals produce viable eggs, but their embryos display severe malformations and high mortality during larval development [34]. Their embryos exhibit reduced levels of long-chain PUFAs, including ω-6 arachidonic acid (ARA) and ω-3 docosahexaenoic acid (DHA), together with increased levels of lipid oxidation products, suggesting that enhanced lipid peroxidation disrupts embryonic lipid homeostasis [35]. A similar depletion of phospholipids and lysophospholipids, particularly those containing DHAs, has also been observed in the brains of adult zebrafish consuming a vitamin E-deficient diet [36]. In mammals, evidence linking lipid metabolism to oxidative stress-mediated NTD remains limited and largely indirect. Early studies in rodents showed that supplementation with ARA can reduce the incidence of NTD and other malformations in embryos exposed to hyperglycemia or anticonvulsant drugs, both ex vivo and in vivo [37,38]. Maternal supplementation with diets enriched in safflower oil, either alone or in combination with vitamin E and inositol, has also been reported to reduce the incidence of diabetic embryopathy in rats [39,40]. More recently, studies on NTD induced by maternal cadmium exposure in mice have implicated altered lipid handling and disrupted lipophagy as factors influencing embryonic development and NTD risk [41]. Despite evidence linking lipid metabolism and embryonic development, the lipid composition of mammalian embryos during early development remains poorly characterized. Most previous studies have focused on the quantification of individual fatty acids using gas chromatography in human and bovine oocytes and embryos [42,43], providing limited information about the diversity of complex lipid species present in embryonic cells.

In this study, we used mass spectrometry-based lipidomics, which enables comprehensive profiling of multiple lipid classes from minimal biological material [44,45] to investigate potential disruptions in lipid homeostasis in SR-B1 KO embryos during neurulation. Given the susceptibility of phospholipids to oxidative damage, we hypothesized that an altered redox balance in SR-B1 KO embryos may lead to enhanced lipid peroxidation and changes in embryonic lipid composition. We applied shotgun lipidomics using a direct-infusion tandem mass spectrometry (DI-MS/MS) approach to analyze lipid profiles in E9.5 mouse embryos. We compared lipid species and categories among WT embryos, SR-B1 KO embryos with normal morphology, and SR-B1 KO embryos exhibiting NTD to identify genotype- and phenotype-associated alterations.

## 2. Materials and Methods

### 2.1. Maintenance of Mice

SR-B1 KO mice, carrying a null mutation in the SR-B1 locus, were maintained on a mixed C57Bl6/J × 129 background (B6;129S2-Scarb1tm1Kri/J) [46]. Animals were kept in plastic cages with nesting material in the animal facility of the Biomedical Research and Innovation Center at Universidad de los Andes at 25 °C and with a 12 h light:dark cycle, and consumed standard chow (Prolab RMH3000, Labdiet, Gray Summit, MO, USA) and water ad libitum. Pregnancies were generated by mating 2- to 4-month-old SR-B1 heterozygous females with 2- to 6-month-old SR-B1 heterozygous males. Female mice were checked daily for the presence of a copulatory plug during the first hour of the light cycle, the detection of which was marked as E0.5. All embryos were collected on day E9.5, when neural tube closure is complete in wild-type embryos.

### 2.2. Retrieval of Embryos and Genotyping

Mice were anesthetized with ketamine (180 mg/kg) and xylazine (12 mg/kg) administered intraperitoneally. The uteri were recovered, and mice were then euthanized by cervical dislocation. Implantation sites were individually retrieved, and embryos and yolk sacs were dissected. Neural tube closure was assessed in embryos, and individual genotyping and sexing were performed using the visceral yolk sac as described [7]. The phenotypic assessments were performed blinded to embryo genotype. The experimental unit was the embryo, although in some experiments, pools of embryos were used to ensure the detection of the molecule of interest. Whole embryos from different litters from the specified genotype (control WT or SR-B1 KO) and phenotype (SR-B1 KO with closed neural tube or NTD) were allocated to the experimental groups. Only embryos with the expected number of somites for E9.5 were used. The experiments were performed using the three experimental groups in parallel, using coding to ensure blinding. The embryos were preserved at −80 °C until use. Embryos used for oxidative stress assays and lipidomics were independent samples.

### 2.3. Thiobarbituric Acid-Reactive Substance (TBARS) Assay

Thiobarbituric acid-reactive substances (TBARS) are a well-recognized parameter of lipid peroxidation. Briefly, pairs of embryos were lysed in 50 µL of RIPA buffer and mixed with thiobarbituric acid (TBA) at a ratio of 1:3.75:1.25 (sample:TBA:H_2_O). TBA was prepared in 0.8% aqueous solution and mixed with 20% acetic acid (pH 3.5) at a 1:1 ratio. The reaction mixture was incubated at 95 °C for 2 h. After cooling, a mixture of n-butanol and pyridine (15:1, *v*/*v*) was added at a ratio of 1:1.25. Samples were gently vortexed and centrifuged at 1000× *g* for 10 min, and the fluorescence of the upper phase was measured (excitation/emission: 530/550 nm) using a Tecan Infinite M1000 PRO reader (Thermo Fisher Scientific, Waltham, MA, USA). Each reaction was performed in duplicate. TBARS levels were determined using a malondialdehyde standard curve and expressed as nmol/mg protein.

### 2.4. Determination of 8-Isoprostane Concentration

The concentration of 8-iso-PGF2α (8-isoprostanes), a representative F2-isoprostane, was measured in lysates (as in Section 2.3) of individual embryos using a competitive ELISA assay (Abcam ab175819, Cambridge, UK). A 96-well plate pre-coated with an anti-8-isoprostane capture antibody was used for the assay. Optical density was recorded at 450 nm using a Tecan Infinite M1000 PRO plate reader (Thermo Fisher, Waltham, MA, USA).

### 2.5. Shotgun Lipidomics

Lipidomic analysis was performed using the service provided by the company Lipotype GmbH (Dresden, Germany) using a shotgun mass spectrometry-based platform as previously described [47]. We used pools of two female embryos from different litters for lipid extraction. Female embryos were selected to avoid variability arising from potential sex-dependent differences detected in different traits during early embryogenesis, even before gonad formation. Intact embryos stored at −80 °C were shipped to Lipotype in dry ice-containing containers.

Homogenates from two whole embryos (in 50 μL) were mixed with ammonium bicarbonate (130 μL), methyl tert-butyl ether/methanol (810 μL; 7:2 *v*/*v*), and 21 μL of a previously prepared internal standards mixture in the same organic solvent. This mixture contained 50 pmol of lysophosphatidylglycerol (LPG) 17:1, 50 pmol of lysophosphatidic acid (LPA) 17:0, 500 pmol of phosphatidylcholine (PC) 17:0/17:0, 30 pmol of hexosylceramide (HexCer) 18:1; 2/12:0, 50 pmol of phosphatidylserine (PS) 17:0/17:0, 50 pmol of phosphatidylglycerol (PG) 17:0/17:0, 50 pmol of phosphatidic acid (PA) 17:0/17:0, 50 pmol of lysophosphatidylinositol (LPI) 17:1, 50 pmol of lysophosphatidylserine (LPS) 17:1, 100 pmol of diacylglycerol (DAG) 17:0/17:0, 50 pmol of triacylglycerol (TAG) 17:0/17:0/17:0, 50 pmol of ceramide (Cer) 18:1; 2/17:0, 200 pmol of sphingomyelin (SM) 18:1; 2/12:0, 50 pmol of lysophosphatidylcholine (LPC) 12:0, 30 pmol of lysophosphatidylethanolamine (LPE) 17:1, 50 pmol of phosphatidylethanolamine (PE) 17:0/17:0, 100 pmol of cholesteryl ester (CE) 20:0, and 50 pmol of phosphatidylinositol (PI) 16:0/16:0. The plate was sealed with a Teflon-coated cover, shaken at 4 °C for 15 min, and centrifuged (3000× *g*, 5 min) to facilitate phase separation. The organic upper phase was cleaned, and 100 μL was transferred to the infusion plate and dried using vacuum centrifugation. Dried lipids were reconstituted in 40 μL of 7.5 mM ammonium acetate in chloroform/methanol/propanol (1:2:4 *v*/*v*/*v*), and the wells were sealed with aluminum foil to prevent evaporation and contamination during infusion. All liquid-handling steps were performed on a Hamilton STARlet robotic platform (Hamilton, Reno, NV, USA) equipped with an anti-drip control for precise organic solvent pipetting.

Direct infusion was performed on a QExactive mass spectrometer (Thermo Fisher Scientific) equipped with a TriVersa NanoMate ion source (Advion Biosciences, Ithaca, NY, USA). Five microliters of the sample was infused with gas pressure and voltage set to 1.25 psi and 0.95 kV, respectively. The delivery time was set to 4 min and 55 s, with a 20 s contact closure delay to prevent aerosol instability. Polarity switching from positive to negative was configured at 135 s after contact closure. Samples were analyzed in both polarities during a single acquisition. In positive ion mode, the acquisition method began by scanning *m*/*z* 402–412 (MS+) at a resolution of R*m*/*z* = 200 = 140,000 to monitor the [Col + NH4^+^]+ ion for 12 s. Each scan segment averaged two micro-scans. The automatic gain control (AGC) target was set to 5 × 10^5^ with a maximum ion injection time (IT) of 200 ms. Next, *m*/*z* 550–1000 was scanned in MS+ mode (R*m*/*z* = 200 = 140,000) with a mass lock on a common background ion (*m*/*z* = 680.48022) for 18 s. The AGC was set to 10^6^ with IT at 50 ms. This was followed by MS/MS+ analysis (R*m*/*z* = 200 = 17,500) using data-independent acquisition triggered by an inclusion list for 105 s. The inclusion list comprised masses from 500.5 to 999.75 in 1 Da intervals. The AGC was set to 10^5^, IT to 64 ms, and the isolation width to 1.0 Da, with the first mass set at 250 Da and normalized collision energy (NCE) at 20%. MS and MS/MS data were combined to monitor CE, DAG, and TAG ions as ammonium adducts. After switching to negative ion mode, a 15 s delay was introduced to allow spray stabilization. *m*/*z* 400–650 was scanned in Fourier Transform Mass Spectrometry in negative ion mode (FTMS^−^) mode (R*m*/*z* = 200 = 140,000) for 15 s, with a mass lock on a common background ion (*m*/*z* = 529.46262) to monitor LPG, LPA, LPI, LPS, and LPE species as deprotonated anions and LPC as an acetate adduct. The AGC was set to 10^6^ and IT to 50 ms. Next, *m*/*z* 520–940 was scanned in (FTMS^−^) mode (R*m*/*z* = 200 = 140,000) for 15 s under the same conditions. Finally, MS/MS- analysis (R*m*/*z* = 200 = 17,500) was performed using data-independent acquisition triggered by an inclusion list for 90 s. This list included masses from 590.5 to 939.5 in 1 Da intervals. The AGC was set to 10^5^, IT to 64 ms, and the isolation width to 1.0 Da, with the first mass at 150 Da and NCE at 35%. MS and MS/MS data were combined to monitor PC, HexCer, Cer, and SM species as acetate adducts and PS, PG, PA, PE, and PI species as deprotonated anions.

For data analysis, all results were processed using Lipotype Zoom, a proprietary lipid identification software developed by the company, based on LipidXplorer 1.2.8.1 [48]. Raw data are available in Table S1.

### 2.6. Quantification of S-Adenosylmethionine (SAM) and S-Adenosylhomocysteine (SAH)

Samples consisting of individual embryos were analyzed in duplicate by liquid chromatography coupled to electrospray ionization tandem mass spectrometry (LC-ESI-MS/MS) as described previously [49].

### 2.7. Lipidomics and Transcriptomics Data Visualization

Analyses at the whole lipidome level were done by data reduction using principal component analysis. Abundance of all detected lipid species (as molar %) was considered in the analysis. The computation was performed in R 4.5.0 with the prcomp() function. In the plot, ellipses indicate the 95% confidence interval and were computed with the factoextra package 2.0.0 To construct volcano plots, we first computed *p*-values and fold changes (as log2) for each individual comparison at the level of lipid species. We used that data as input and rendered the final plot with the EnhancedVolcanoPlot package 1.26.0 in R. When presenting individual values of selected lipid species or genes, we used heatmaps. Lipid species were selected by filtering the full database to remove all lipids with molar % below the 10th percentile (this value corresponded to 0.004%mol) and leaving only those species with a statistically significant difference in at least one comparison. Selected species were separated by class and plotted in heatmaps, showing the z-score of molar %.

For transcriptomics analysis, we selected genes involved in fatty acid remodeling based on Gene Ontology terms and extracted their expression levels from our previously described sequencing data from WT, SR-B1 KO and SR-B1 KO NTD embryos at E9.5 [50]. We plotted a z-score of normalized expression (by the DESeq2 1.14.2 procedure) of all selected genes and indicated differentially expressed genes with dotted boxes.

Supplementary Figure S1 generated using ChatGPTversion GPT-5.5 (OpenAI) shows a schematic representation of the main integrated lipidomic and transcriptomic changes detected in SR-B1 KO NTD embryos relative to WT embryos. This image was based on author-provided input and was reviewed and edited for scientific accuracy by the authors.

### 2.8. Statistics

Results are presented as mean ± SEM. For each variable, the normality of the distributions was assessed using the Kolmogorov–Smirnov test. Depending on the variables analyzed, either one-way or two-way ANOVA was applied. Post hoc analyses were performed as indicated in the corresponding figures. The statistical analysis was performed using GraphPad Prism (version 8, GraphPad Software, San Diego, CA, USA), and differences were considered statistically significant at *p* < 0.05. 

## 3. Results

### 3.1. Detection of Lipoperoxidative Markers in WT and SR-B1 KO Embryos

**Figure 1:**
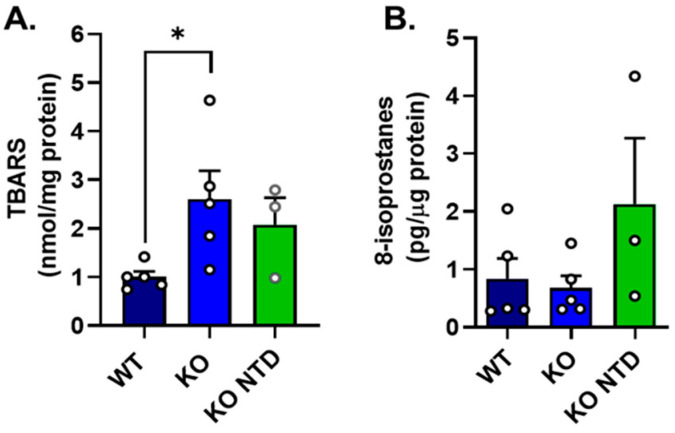
Lipoperoxidation markers in WT, SR-B1 KO (KO), and SR-B1 KO NTD (KO NTD) embryos. (**A**) Thiobarbituric acid-reactive substances (TBARSs) and (**B**) 8-isoprostanes levels, measured in WT, SR-B1 KO, and SR-B1 KO NTD embryos. Each point represents a pool of two embryos for the TBARS assay and an individual sample for 8-isoprostanes. Bars indicate mean ± SEM. Statistical significance is indicated as * *p* < 0.05. Data were analyzed by one-way ANOVA with Dunn’s post hoc test.

Our previous studies demonstrated that SR-B1 KO embryos, compared to their wild-type (WT) littermates, were severely vitamin E deficient and showed increased levels of ROS [11], suggesting an altered redox status in those embryos. In the current study, oxidative lipid damage markers were compared between WT and SR-B1-deficient embryos with normal morphology (SR-B1 KO) or with NTD (SR-B1 KO-NTD). Lipid peroxidation markers showed distinct patterns among experimental groups. Compared to WT embryos, SR-B1 KO embryos without NTD exhibited higher mean levels of thiobarbituric acid-reactive substances (TBARSs) (Figure 1A). SR-B1 KO with NTD also showed an elevated TBARS concentration, but this difference did not reach significance.

By contrast, 8-isoprostane levels were similar between WT and SR-B1 KO embryos without NTD, while SR-B1 KO embryos presenting NTD displayed a tendency to higher values than the other two groups (Figure 1B). Overall, these findings indicate that SR-B1 deficiency is associated with increased oxidative lipid damage during embryonic development, although the relationship with embryo phenotype differs depending on the marker evaluated and is accompanied by substantial inter-embryo variability.

### 3.2. Lipidomic Profiles in WT and SR-B1 KO Embryos

**Figure 2:**
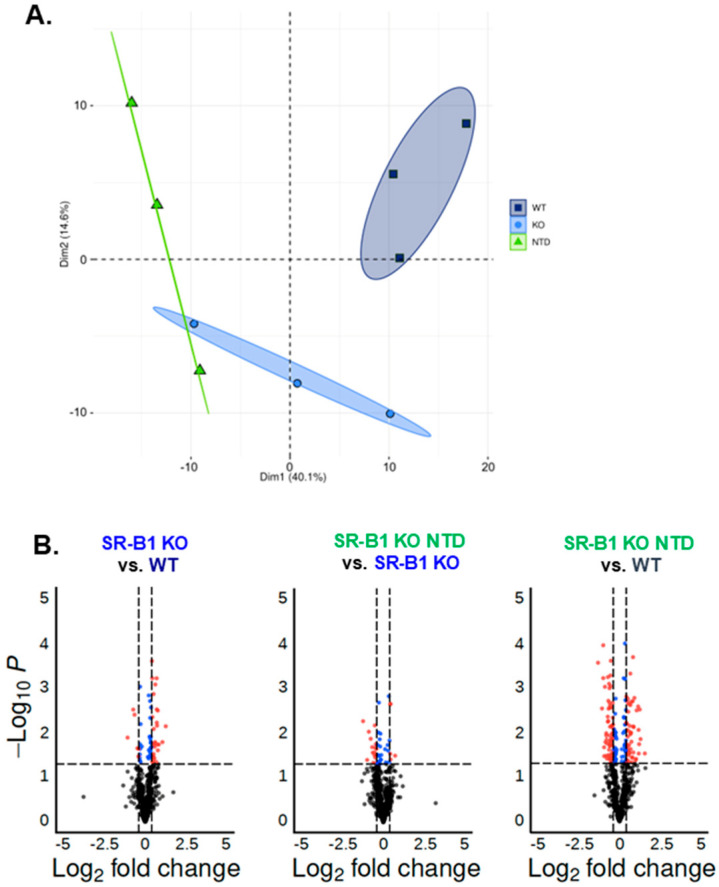
Principal component and pairwise differential lipidomic analysis in WT, SR-B1 KO, and SR-B1 KO NTD embryos. (**A**) Principal component analysis showing separation of lipidomic profiles among WT, SR-B1 KO, and SR-B1 KO NTD embryos. Each point represents an individual sample, and ellipses indicate group clustering. (**B**) Volcano plots showing pairwise comparisons between groups. The *x*-axis represents log_2_ fold change, and the *y*-axis represents −log_10_ *p*-value. Dashed lines indicate significance thresholds (*p* < 0.05 and log_2_ fold change >50% cutoff as indicated). Differentially abundant lipid species are highlighted, with red dots representing significantly increased species, blue dots representing significantly decreased species, and black dots representing non-significant species.

We next evaluated whether the altered redox status and lipoperoxidative damage detected in neurulating SR-B1 KO embryos were associated with changes in their lipid composition. Shotgun lipidomics enabled the detection of more than 20 lipid classes with information on their fatty acid composition and unsaturation. Principal component analysis revealed distinct clustering patterns among the three groups of embryos (Figure 2A). WT embryos exhibited a lipidomic profile distinct from that of SR-B1 KO NTD embryos, while normal SR-B1 KO embryos displayed an intermediate fingerprint, only partially overlapping with the NTD group, showing that the lipid profile can readily distinguish the three experimental groups.

We performed the following pairwise comparisons in volcano plot analyses: (1) SR-B1 KO vs. WT, (2) SR-B1 KO NTD vs. SR-B1 KO, and (3) SR-B1 KO NTD vs. WT (Figure 2B). The number of species displaying differences in WT vs. SR-B1 KO (*n* = 34) was higher than in SR-B1 KO vs. SR-B1 KO NTD (*n* = 21), suggesting that genotype is more predictive of changes in lipid content than the NTD morphology alone. However, the highest number of altered lipids was found in the WT vs. SR-B1 KO NTD comparison (*n* = 112), suggesting a synergistic interaction between genotype and phenotype, resulting in an enhanced oxidative stress–driven impact on embryonic lipid composition.

**Figure 3:**
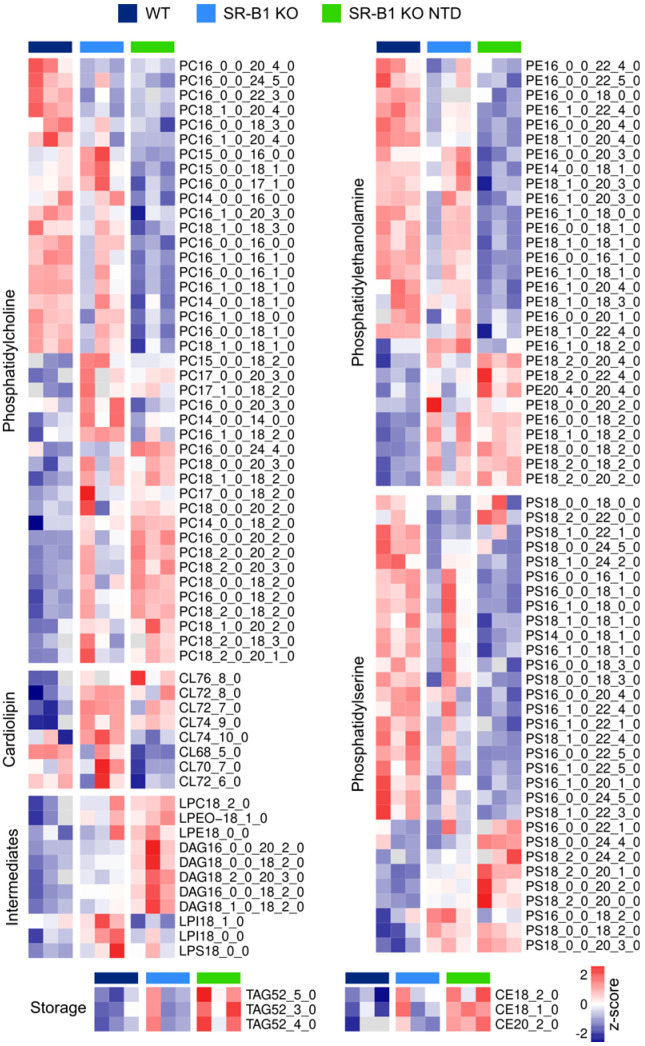
Lipidomic profiling in WT, normal SR-B1 KO, and SR-B1 KO NTD embryos. Heatmaps showing Z-score normalized relative abundances of lipid species across experimental groups. Lipid classes are organized by panels, including phosphatidylcholine (PC), phosphatidylethanolamine (PE), phosphatidylserine (PS), cardiolipin (CL), intermediates (lysophospholipids including LPC, LPE, LPI, and LPS, as well as diacylglycerol [DAG]), and storage lipids (triglycerides [TAGs] and cholesterol esters [CEs]). The color scale indicates relative abundance, with red representing higher and blue lower levels compared to the mean across all samples. Distinct clustering patterns highlight alterations in lipid composition associated with SR-B1 deficiency and the presence of NTD.

Exploration of these differences at the single species level revealed coordinated remodeling of multiple phospholipid classes in SR-B1-KO embryos (Figure 3). Phospholipid species from different classes (phosphatidylcholine, phosphatidylethanolamine, and phosphatidylserine) showed marked trends: SR-B1 KO NTD samples had extreme changes -either up or downwards-, whereas SR-B1 KO embryos showed intermediate levels. In contrast, some storage lipids had a clear signature, where SR-B1 KO NTD embryos accumulated more of these classes of lipids. Notably, triacylglycerides (TAGs) and cholesteryl esters (CEs) containing PUFAs were among the more abundant species in SR-B1 KO NTD embryos. Among TAGs, some species such as TAG(52:5), TAG(52:3), and TAG(52:4), were markedly increased in SR-B1 KO NTD embryos compared to WT and SR-B1 KO. In parallel, cholesteryl esters (CEs), particularly CE(18:2), CE(18:1), and CE(20:2), also displayed elevated levels in SR-B1 KO NTD embryos. These general changes in lipid species suggest selective remodeling of fatty acid membrane composition and storage compartments.

### 3.3. Genotype- and Phenotype-Associated Differences in Fatty Acids in Phospholipids of SR-B1 KO Embryos

To determine whether SR-B1 deficiency was associated with broader remodeling of phospholipid fatty acid composition beyond changes detected at the individual molecular species level, we examined the overall distribution of ω3 and ω6 PUFAs within embryonic phospholipids. We calculated the total amounts of specific ω3 and ω6 PUFAs within each phospholipid class by adding all individual subspecies containing the respective PUFAs in their fatty acid chains. This approach allowed us to identify broader patterns that may not have been detected by more granular analyses.

**Figure 4:**
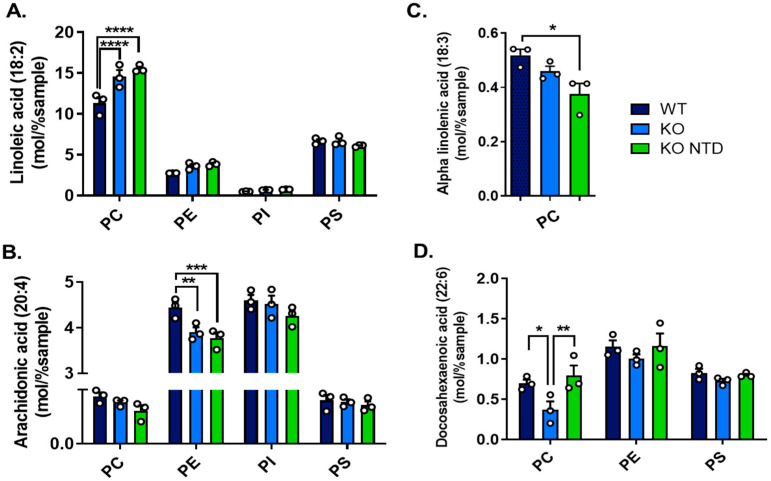
ω-3 and ω-6 PUFA content across phospholipid classes in WT, SR-B1 KO (KO), and SR-B1 KO NTD (KO NTD) embryos. (**A**) Linoleic acid (18:2, ω-6), (**B**) arachidonic acid (20:4, ω-6), (**C**) α-linolenic acid (18:3, ω-3) and (**D**) docosahexaenoic acid (22:6, ω-3) levels in different phospholipid classes. Data are expressed as mol% of total lipids. Bars represent mean ± SEM. Statistical significance is indicated as follows: * *p* < 0.05; ** *p* < 0.01; *** *p* < 0.001; **** *p* < 0.0001. Panels (**A**,**B**,**D**) were analyzed by two-way ANOVA, and panel (**C**) was analyzed by one-way ANOVA, with Tukey’s post hoc tests. PC, phosphatidylcholine; PE, phosphatidylethanolamine; PI, phosphatidylinositol; and PS, phosphatidylserine.

We compared the relative total amounts of arachidonic acid (ARA, 20:4, ω6), docosahexaenoic acid (DHA, 22:6, ω3), and their essential precursors, linoleic acid (LA, 18:2, ω6) and α-linolenic acid (ALA, 18:3, ω3), in phospholipids. These PUFAs were selected due to their diverse cellular roles, including gene regulation, membrane fluidity and function, and as precursors of various bioactive lipids [27]. We also aimed to include eicosapentaenoic acid (EPA, 20:5, ω3); however, this lipid was either undetectable or present at extremely low levels. Compared to WT embryos, both morphologically normal SR-B1 KO embryos and SR-B1 KO with NTD embryos displayed increased LA levels in phosphatidylcholine (PC) and decreased ARA levels in phosphatidylethanolamine (PE) (Figure 4A,B). ALA levels were only detected in PC, where a significant reduction was observed in SR-B1 KO embryos with NTD in comparison to WT embryos (Figure 4C). DHA levels varied according to embryonic morphology: SR-B1 KO embryos with normal morphology had reduced DHA, while NTD embryos exhibited normal levels of this lipid (Figure 4D). These differences in DHA were only significant in PC, although the same tendency was observed in PE and PS. Together, these results indicate selective remodeling of essential and long-chain PUFA in embryonic phospholipids associated with SR-B1 deficiency, particularly affecting PC and PE, with genotype-related changes in ω6-containing phospholipids and phenotype-dependent differences in ω3 distribution.

### 3.4. Disruption of Saturated and Monounsaturated (MUFA) Profiles in Phospholipids Associated with the NTD Phenotype

**Figure 5:**
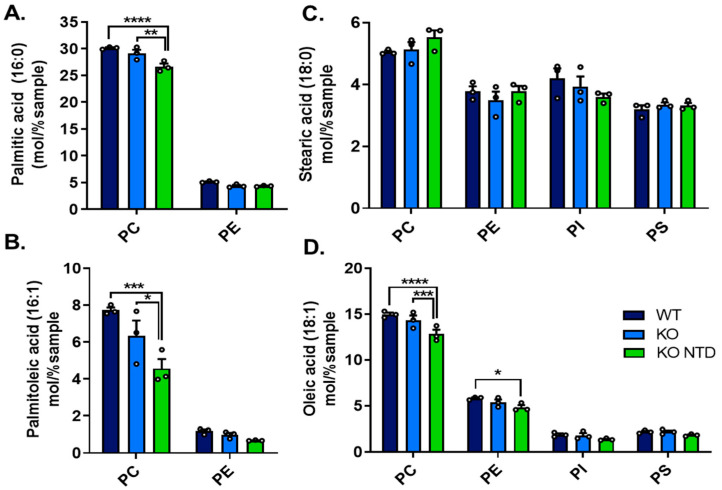
Saturated and monounsaturated fatty acid content across phospholipid classes in WT, SR-B1 KO (KO), and SR-B1 KO NTD (KO NTD) embryos. (**A**) Palmitic acid (16:0), (**B**) palmitoleic acid (16:1, ω-7), (**C**) stearic acid (18:0), and (**D**) oleic acid (18:1, ω-9) levels in different phospholipid classes (PC, PE, PI, and PS). Data are expressed as mol% of total lipids. Bars represent mean ± SEM, and each point corresponds to an individual sample. Statistical significance is indicated as follows: * *p* < 0.05; ** *p* < 0.01; *** *p* < 0.001; and **** *p* < 0.0001. Data were analyzed by two-way ANOVA with Tukey’s post hoc test. PC, phosphatidylcholine; PE, phosphatidylethanolamine; PI, phosphatidylinositol; and PS, phosphatidylserine.

Analysis of saturated fatty acids across different phospholipid classes showed that the content of these lipids was similar in SR-B1 KO embryos with a closed neural tube than in WT embryos. By contrast, SR-B1 KO embryos with NTD contained lower levels of palmitic acid in PC (Figure 5A), whereas stearic acid levels were unchanged (Figure 5C). Regarding MUFA species, no changes were detected between WT and SR-B1 KO embryos with normal morphology, but the NTD phenotype was associated with lower palmitoleic levels in PC and decreased oleic acid levels in PC and PE (Figure 5B,D). Together, these results suggest altered levels of specific ω9-related MUFA in distinct phospholipid classes, mainly associated with the NTD phenotype, possibly reflecting subtle changes in fatty acid elongation and/or desaturation pathways involved in the synthesis of palmitoleic and oleic acid.

### 3.5. Increased Lysophosphatidylcholine-to-Phosphatidylcholine Ratio in SR-B1 KO Embryos

**Figure 6:**
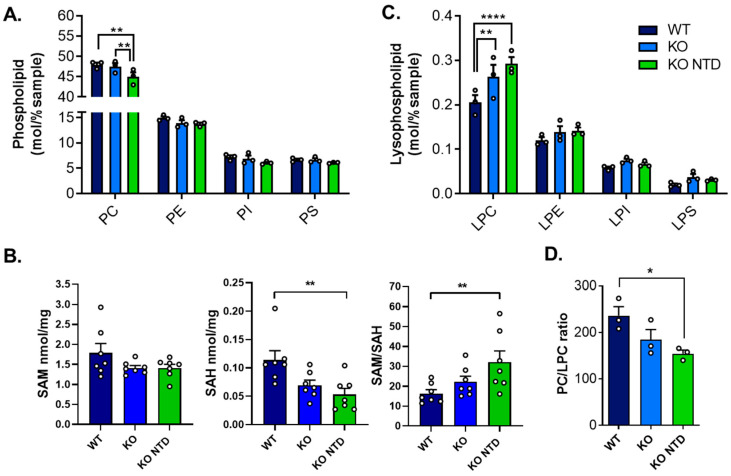
Phospholipid and lysophospholipid composition and methionine cycle metabolites in WT, SR-B1 KO (KO), and SR-B1 KO NTD (KO NTD) embryos. (**A**) Phospholipid class distribution (PC, PE, PI, and PS) and (**C**) lysophospholipid levels (LPC, LPE, LPI, and LPS), expressed as mol% of total lipids. (**B**) S-adenosylmethionine (SAM), S-adenosylhomocysteine (SAH) levels (nmol/mg) and SAM/SAH ratio. (**D**) PC/LPC ratio. Bars represent mean ± SEM, and each point corresponds to an individual sample. Statistical significance is indicated as follows: * *p* < 0.05; ** *p* < 0.01; and **** *p* < 0.0001. Data were analyzed by two-way ANOVA (**A**,**C**) or one-way ANOVA (**B**,**D**) with Tukey’s post hoc test. PC, phosphatidylcholine; PE, phosphatidylethanolamine; PI, phosphatidylinositol; PS, phosphatidylserine; LPC: lysophosphatidylcholine; LPE: lysophosphatidylethanolamine; LPI: lysophosphatidylinositol; and LPS: lysophosphatidylserine.

To determine whether the fatty acid remodeling observed in SR-B1-deficient embryos was accompanied by broader alterations in membrane phospholipid homeostasis, we next examined total phospholipid class abundance across embryos from the various groups. Compared to WT embryos, SR-B1 KO embryos with NTD—and not SR-B1 KO embryos with closed neural tubes—displayed lower total PC levels, while other phospholipids remained unaffected (Figure 6A). We evaluated whether the reduction in PC levels in SR-B1 KO embryos was associated with alterations in the availability of S-adenosylmethionine (SAM), a metabolite of the 1-carbon cycle required for the synthesis of PC through the canonical phosphatidylethanolamine N-methyltransferase (PEMT) [51]. Oxidative stress can impair methionine adenosyltransferase (MAT) activity, the enzyme that synthesizes SAM, leading to reduced SAM levels [52]. We measured SAM and its metabolite S-adenosylhomocysteine (SAH) and calculated the SAM/SAH ratio in individual embryos. The mean levels of SAM were 20% less in SR-B1 KO embryos independently of their morphology, but this difference did not reach statistical significance (Figure 6B). By contrast, SAH levels were significantly reduced in SR-B1 KO embryos with NTD compared with WT embryos, whereas SR-B1 KO embryos showed intermediate values. As a result, the SAM/SAH ratio differed significantly among groups, with SR-B1 KO NTD embryos displaying higher ratios compared with WT embryos, although this difference was primarily driven by the reduction in SAH. To determine whether the reduction in PC resulted from an increase in hydrolysis catalyzed by phospholipases, we analyzed lysophospholipid (LysoPLs) levels. SR-B1 deficiency was associated with elevated lysophosphatidylcholine (LPC) levels in embryos of both phenotypes (Figure 6C). Consistent with this finding, SR-B1 KO embryos with NTD exhibited a significantly lower PC/LPC ratio compared to WT embryos (Figure 6D). Overall, these results indicate that the reduction in PC abundance observed in SR-B1 KO embryos is not associated with a severe limitation in SAM availability and correlates with an increase in LPC content. The combined reduction in selected phosphatidylcholine species and increase in LPC suggest enhanced phospholipid turnover or hydrolysis in SR-B1-deficient embryos.

### 3.6. Elevated Storage Lipids in SR-B1 KO Embryos Presenting NTD

**Figure 7:**
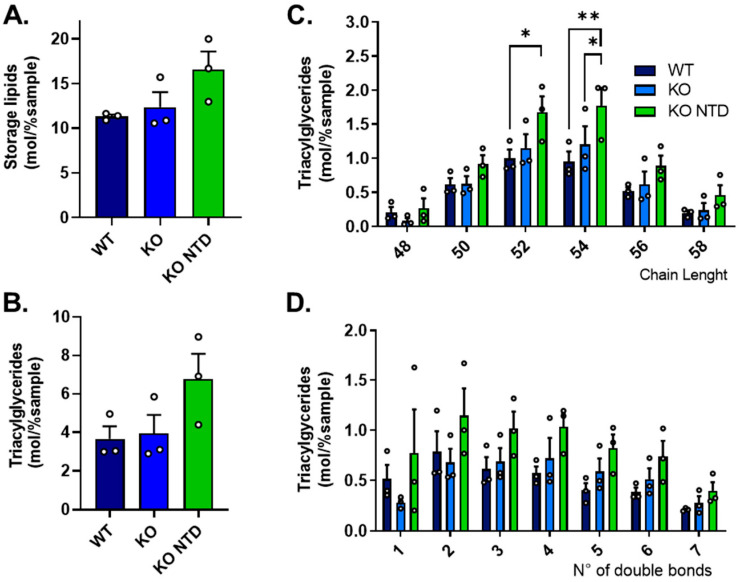
Storage lipid and triacylglyceride content in WT, SR-B1 KO (KO), and SR-B1 KO NTD (KO NTD) embryos. (**A**) Storage lipid and (**B**) Total triacylglyceride (TAG) levels (mol% per sample). (**C**) TAG species distribution according to the number of double bonds and (**D**) TAG species distribution according to acyl chain length. Bars represent mean ± SEM, and individual data points are shown. Statistical significance is indicated as follows: * *p* < 0.05 and ** *p* < 0.01. Panels (**A**,**B**) were analyzed by one-way ANOVA and Dunns post hoc test, and panels (**C**,**D**) were analyzed by two-way ANOVA and Tukey’s post hoc test.

We assessed whether phospholipid remodeling in SR-B1-deficient embryos was accompanied by redistribution of fatty acids toward neutral lipid storage, and found no significant differences, although a clear tendency towards higher storage lipid levels was observed in SR-B1 KO NTD embryos, and not in morphologically normal SR-B1 KO embryos, when compared to WT samples (Figure 7A). This tendency was primarily driven by TAGs (Figure 7B). TAG accumulation in SR-B1 KO NTD embryos was broadly distributed across species and not restricted to a specific chain length, although TAG species containing fatty acids with a combined total of 52 and 54 carbon atoms were significantly elevated compared to WT embryos (Figure 7C). Although no significant differences in TAG accumulation were observed based on the degree of unsaturation, TAGs with more than two double bonds consistently tended to be more abundant in SR-B1-deficient embryos, particularly in those with NTD (Figure 7D). Together, these results suggest that SR-B1 KO embryos with NTD accumulate specific TAG species and tend to store more unsaturated fatty acids, suggestive of altered fatty acid partitioning toward neutral lipid storage.

### 3.7. Altered Expression of Genes Involved in Lipogenesis and Phospholipid Remodeling in SR-B1 Deficient Embryos

**Figure 8:**
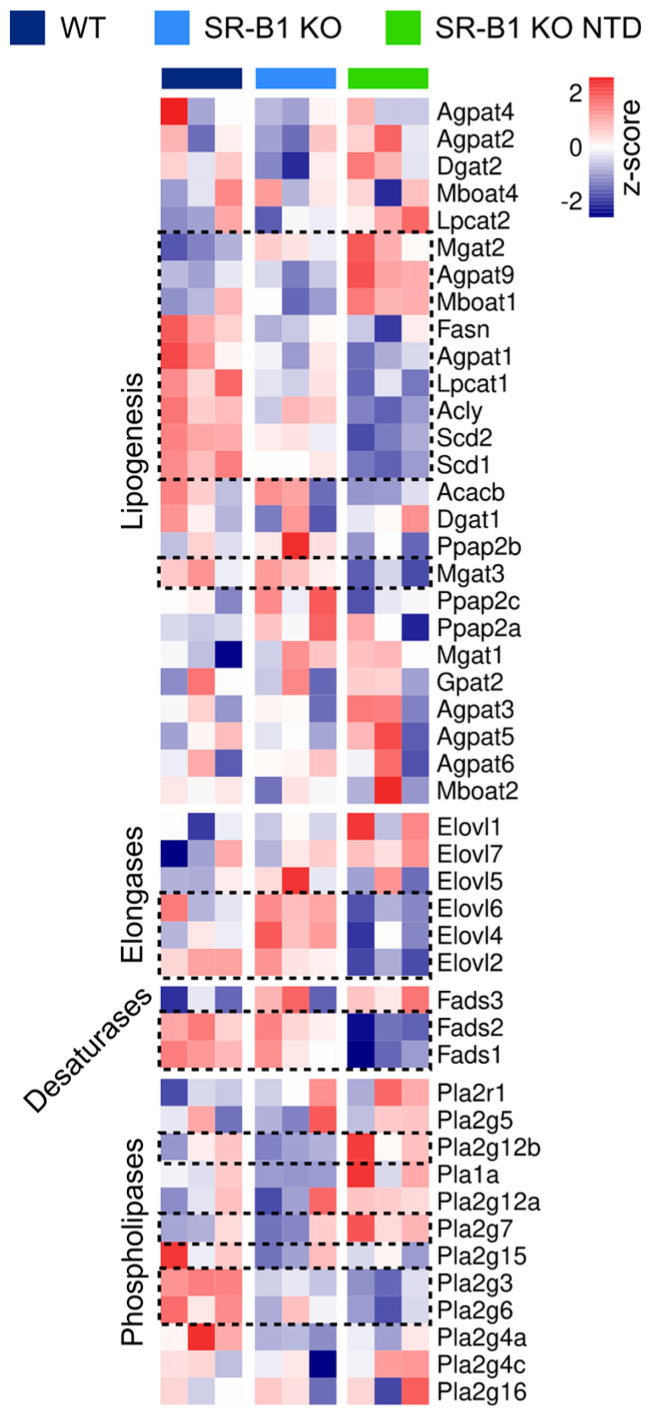
Differential expression of genes involved in fatty acid synthesis and remodeling in WT, SR-B1 KO, and SR-B1 KO NTD embryos. Heatmap representation of RNA-seq data showing the relative expression (z-score) of genes involved in lipogenesis and fatty acid remodeling, selected using Gene Ontology (GO) terms. Each column represents an individual sample, and each row corresponds to a gene. Gene expression values were normalized and scaled across samples. The color scale ranges from low (blue) to high (red) expression. Dotted boxes indicate genes identified as differentially expressed.

To determine whether the lipidomic alterations observed in SR-B1-deficient embryos were associated with transcriptional changes in lipid metabolic pathways, we used our previously generated RNA sequencing database for WT, SR-B1 KO, and SR-B1 KO NTD embryos [50] and analyzed the expression of genes involved in lipogenesis, fatty acid elongation and desaturation, and phospholipid hydrolysis. As observed in Figure 8, showing the relative expression of genes in selected functional categories, SR-B1 KO embryos with NTD displayed the most distinct expression profile.

Within the lipogenesis module, SR-B1 KO NTD embryos showed a general reduction in the expression of genes involved in de novo lipid synthesis, including fatty acid synthase (Fasn), ATP citrate lyase (Acly), and stearoyl-CoA desaturase 1 (Scd1), consistent with a decreased capacity for fatty acid synthesis and monounsaturated fatty acid (MUFA) production. In contrast, genes associated with lipid remodeling and acyltransferase activity, such as lysophosphatidylcholine acyltransferase 2 (Lpcat2), monoacylglycerol acyltransferase 2 (Mgat2), and 1-acylglycerol-3-phosphate O-acyltransferase 9 (Agpat9), were increased, indicating enhanced phospholipid turnover and glycerolipid remodeling. In the elongases module, elongation of very long-chain fatty acids protein 2 (Elovl2), Elovl4, and Elovl6 showed decreased expression in SR-B1 KO NTD embryos relative to WT, suggesting a reduced elongation capacity for long-chain fatty acids. In the desaturase module, expression of fatty acid desaturase 1 (Fads1) and Fads2 was reduced, consistent with impaired desaturation of polyunsaturated fatty acids. Finally, within the phospholipase module, several genes involved in phospholipid hydrolysis were upregulated, including phospholipase A2 group XIIB (Pla2g12b), supporting increased phospholipid remodeling.

## 4. Discussion

Although oxidative stress has been proposed as a common etiological factor in NTD due to diverse environmental exposures, its impact on embryonic lipids during mammalian neurulation remains largely unexplored. The evidence presented in this study shows higher lipoperoxidation in SR-B1-deficient embryos. Although the increase is only significant in TBARSs for non-malformed SR-B1 KO embryos, SR-B1 embryos with NTD show a tendency to both higher levels of TBARSs and 8-isoprostanes. Surprisingly, SR-B1 KO embryos with normal morphology show similar levels of 8-isoprostanes to WT embryos. These apparent discrepancies in the levels of the two lipoperoxidation markers evaluated probably arise from the fact that they measure different molecules: TBARSs estimate malondialdehyde (MDA), a secondary product of polyunsaturated fatty acid peroxidation, although it may also detect other lipid peroxidation products, whereas 8-isoprostanes are prostaglandin-like compounds formed non-enzymatically from arachidonic acid and are considered among the most specific and reliable biomarkers of lipid peroxidation in vivo. Further studies using additional markers of lipoperoxidative damage may be needed to understand the biological implications of these results.

This study also shows selective modifications in embryonic lipids in neurulating SR-B1 KO mouse embryos exhibiting ROS linked to vitamin E deficiency [11]. This remodeling is characterized by altered phospholipid fatty acid composition and is accompanied by increased LPC and an accumulation of some species of TAG. One interesting observation from our results is that SR-B1 KO embryos presenting NTD exhibit more pronounced alterations in lipids, whereas morphologically normal SR-B1 KO embryos show an intermediate lipidomic profile. These results suggest that SR-B1 deficiency may establish a basal disturbance in embryonic lipid homeostasis that, together with embryo-to-embryo variability in transcriptional and metabolic responses [50], contributes to an imbalance that exceeds the critical threshold to cause NTD in some embryos and not in others. This idea is consistent with the incomplete penetrance of NTD in embryos lacking SR-B1 [7].

Our results do not show a generalized effect of SR-B1 deficiency on PUFA content. Although ARA is less abundant in selected phospholipids in SR-B1 KO embryos with NTD, DHA content is preserved, and LA content is increased, indicating selective remodeling rather than global PUFA depletion. Reduced levels of ARA in embryonic phospholipids may have developmental consequences because this fatty acid has been proposed to contribute to membrane organization and serve as a precursor for prostaglandins and other bioactive lipids required during neurulation [37,53]. The simultaneous increase in LA together with reduced expression of Fads1 and Fads2 suggests impaired conversion of precursor ω6 fatty acids into long-chain derivatives, which may limit ARA availability in SR-B1 KO embryos with NTD. Consistent with this interpretation, previous studies have shown that ARA supplementation reduces neural tube defect incidence in experimental models of oxidative stress-associated embryopathy [38,54].

An interesting finding was that SR-B1 KO embryos with NTD exhibit reduced total PC levels. This phospholipid is synthesized via two pathways: the cytidine diphosphate (CDP)–choline (Kennedy) pathway, which mainly produces PC species containing mono- and di-unsaturated fatty acids, and the PEMT pathway, which uses SAM to methylate phosphatidylethanolamine, preferentially generating PUFA-enriched PC species such as PC-DHA [55]. The results of our study suggest that the availability of methyl groups for remethylating homocysteine is not severely affected in SR-B1 with NTD. However, the reduced abundance of both SAM and SAH indicates a potentially altered availability or increased turnover of methionine cycle intermediates. A more rigorous metabolic analysis would be required to better understand the alterations in one-carbon metabolism in SR-B1 KO embryos. Although the PEMT pathway has been proposed as a source of DHA-containing PC in certain tissues such as the liver and adipose tissue [56], PEMT activity is not detectable in neurulating mouse embryos in culture [57], so it is likely that PC synthesis at this stage relies predominantly on the CDP-choline pathway.

The increase in LPC concomitant with the reduction in PC supports enhanced phospholipid hydrolysis in SR-B1 KO embryos with NTD. PC can be hydrolyzed by PLA or directly through ROS-mediated cleavage. The most extensively studied PLA is cPLA2, and its activation has generally been shown to cause the preferential release of ARA from phospholipids [58]. In our work, ARA levels were reduced in KO embryos with NTD, supporting the hypothesis that this mechanism may be operating. LPC accumulation is a well-recognized feature of oxidative stress and reflects the removal of oxidized fatty acyl chains from membrane phospholipids, a process that contributes to membrane repair [27]. LPC enrichment can alter the plasma membrane biophysical properties and influence signaling pathways involved in processes relevant to neural fold elevation and fusion, such as cell polarity and morphogenetic movements.

SR-B1 KO embryos with NTD also show decreased abundance of multiple PC species containing distinct fatty acids, compared to WT embryos and morphologically normal SR-B1 KO embryos. Analysis of fatty acid composition within PC revealed a selective remodeling pattern, characterized by increased LA in SR-B1-deficient embryos and altered distribution of DHA depending on embryonic phenotype. Elevated LA levels together with decreased ALA levels in PC in SR-B1 KO embryos with NTD may have biological relevance. First, studies on neural stem cell behavior in vitro have demonstrated that the ALA/LA ratio modulates their proliferation and differentiation [59,60], although the specific role of each of these lipids in neurulation has still not been described. Also, these essential fatty acids are precursors of ARA and DHA, which play critical roles during neural development by regulating neural stem/progenitor cell proliferation, differentiation, and neurogenesis through modulating membrane composition and production of bioactive lipids [61,62]. DHA levels in PC were lower in morphologically normal SR-B1 KO embryos than in WT embryos. A depletion of DHA-containing phospholipids was reported previously in zebrafish embryos spawned from females fed vitamin E-deficient diets [63]. Unexpectedly, the content of PC-DHA in SR-B1 KO embryos with NTD was preserved, suggesting that compensatory mechanisms aimed at maintaining critical polyunsaturated fatty acids may be operating. Rather than preventing the abnormal phenotype, DHA preservation may reflect an incomplete adaptive response that is insufficient to restore the complex membrane dynamics and signaling processes necessary for normal neurulation.

The reduction in MUFA observed in SR-B1 KO embryos with NTD may have important consequences for membrane homeostasis and susceptibility to oxidative damage. MUFAs contribute to membrane fluidity but are less susceptible to peroxidation than PUFAs [64]. In this context, decreased levels of oleic and palmitoleic acid may reduce the ability of embryos to maintain optimal membrane biophysical properties during neurulation. Recent evidence has demonstrated that enrichment in MUFA protects cells from ferroptosis, a form of cell death driven by iron-dependent lipid peroxidation [65,66]. Therefore, the depletion of MUFAs in SR-B1-deficient embryos may further exacerbate lipid peroxidation and compromise membrane integrity, contributing to defective morphogenetic processes required for neural tube closure.

SR-B1 KO embryos with NTD exhibit a clear tendency to accumulate storage lipids, primarily TAG, compared to WT embryos. This result is unlikely to represent a simple increase in energy storage, but rather an adaptive response to oxidative stress, because lipid droplet biogenesis is an adaptive mechanism against cellular oxidative stress [67]. Long considered merely an energy reservoir, lipid droplets have recently emerged as structures capable of performing diverse crucial tasks to protect cellular integrity under stress: they sequester potentially toxic lipids and proteins, maintain redox and energy balance, preserve membrane and organelle homeostasis, and provide lipids that act as signaling mediators [68,69,70]. One of the strongest pieces of evidence for the role of lipid droplets as organelles involved in redox homeostasis comes from Drosophila neurodevelopment [71]. In response to increased ROS, neuroblasts and glial cells activate lipid droplet formation and redistribute fatty acids from membrane phospholipids to TAG as a strategy to prevent lipoperoxidative damage. Thus, SR-B1 KO embryos with NTD potentially exhibit increased mobilization of fatty acids into TAG as an adaptive mechanism to prevent oxidative and/or inflammatory damage to membrane lipids.

Despite the above insights, several limitations of this study should be acknowledged when interpreting the results. First, we analyzed E9.5 embryos, at which stage neural tube closure is complete. Although soon after closure failure in NTD, this limits causal inference, as we cannot rule out the possibility that differences observed between SR-B1 KO embryos with or without NTD reflect secondary consequences of the established phenotype. Second, we used pooled, female embryos for the analysis, so our results may not fully capture biological variability between individual embryos or potential sex-dependent differences. Studies including both male and female embryos will be necessary to determine whether sex-dependent differences in susceptibility to lipid peroxidation contribute to the higher incidence of NTD observed in females. Third, the link between oxidative stress and lipid remodeling is based on correlative lipidomic and transcriptomic data. Future functional experiments may confirm whether these alterations are causally involved in the development of the phenotype. Indeed, impaired lipid handling in SR-B1 KO extraembryonic tissues may also contribute to the disruptions, as SR-B1 and other lipoprotein receptors expressed in these tissues are required for the uptake of maternal lipoprotein-derived lipids during neurulation [72,73]. For instance, reduced MUFA uptake due to abnormal lipid processing in the SR-B1 KO yolk sac may exacerbate oxidative stress, establishing a feedback loop that amplifies lipid alterations and promotes the pathological outcome.

To conclude, our findings identify lipoperoxidation, phospholipid remodeling and neutral lipid accumulation as embryonic disruptions that may be associated with increased oxidative stress during neurulation in SR-B1-deficient embryos. Rather than a generalized depletion of lipids, our study demonstrates a selective and coordinated reorganization of fatty acid distribution, characterized by altered PUFA and MUFA composition, increased LPC levels, and diversion of fatty acids into TAG storage. Figure S1 shows a schematic representation of the main lipidomic and transcriptomic changes detected in SR-B1 KO NTD embryos relative to WT embryos. This integrated response suggests the activation of adaptive mechanisms aimed at preserving membrane integrity and limiting lipid peroxidation. In some of the embryos, this response may result in insufficient capacity to sustain neural tube closure, consistent with the incomplete penetrance of NTD in SR-B1 KO embryos. 

Together, the results of the present work provide novel information showing that oxidative stress may disrupt mammalian embryonic lipid metabolism, highlighting lipid remodeling as a potential contributor to susceptibility to NTD. These findings open new avenues for understanding the interplay between antioxidant status and lipid homeostasis in early development and suggest that targeting lipid metabolism, in addition to redox balance, may represent a novel and promising strategy for the prevention of human NTD.

## Data Availability

The original contributions presented in this study are included in the Supplementary Material. Further inquires can be directed to the corresponding author. The transcriptomic data generated in a previous study and analyzed in this paper are publicly available in the Gene Expression Omnibus (GEO) repository under accession number GSE115091.

## Supplementary Materials

The following supporting information can be downloaded at: https://www.mdpi.com/article/10.3390/antiox15050634/s1. Figure S1: Schematic representation of integrated main lipidomic and transcriptomic changes in SR-B1 KO NTD embryos relative to WT embryos; Table S1: Lipid species in embryonic extracts normalized to the total sample amount (mol%).

## Abbreviations

ARA	Arachidonic acid
ALA	α-Linolenic acid
CE	Cholesteryl ester
DAG	Diacylglycerol
DHA	Docosahexaenoic acid
LA	Linoleic acid
LPA	Lysophosphatidic acid
LPC	Lysophosphatidylcholine
LPE	Lysophosphatidylethanolamine
LPI	Lysophosphatidylinositol
LPS	Lysophosphatidylserine
MUFAs	Monounsaturated fatty acids
NTD	Neural tube defects
OA	Oleic acid
PC	Phosphatidylcholine
PE	Phosphatidylethanolamine
PEMT	Phosphatidylethanolamine N-methyltransferase
PI	Phosphatidylinositol
PLA	Phospholipase A
PS	Phosphatidylserine
PUFAs	Polyunsaturated fatty acids
pYS	Parietal yolk sac
ROS	Reactive oxygen species
SAH	Adenosylhomocysteine
SAM	S-adenosylmethionine
SEM	Standard error of the mean
SM	Sphingomyelin
SR-B1	Scavenger Receptor class B type 1
TAGs	Triacylglycerides
TBARSs	Thiobarbituric acid-reactive substances
WT	Wild type
