# Supplementary material for: Lipid Remodeling in Mouse SR-B1-Deficient Embryos with Oxidative Stress-Associated Neural Tube Defects
Source: Antioxidants (Basel). 2026 May 16;15(5):634. doi: 10.3390/antiox15050634 (PMC13203163; doi:10.3390/antiox15050634)
Supplement: Supplementary file 1 [file antioxidants-15-00634-s001.zip › Figure S1.pdf]

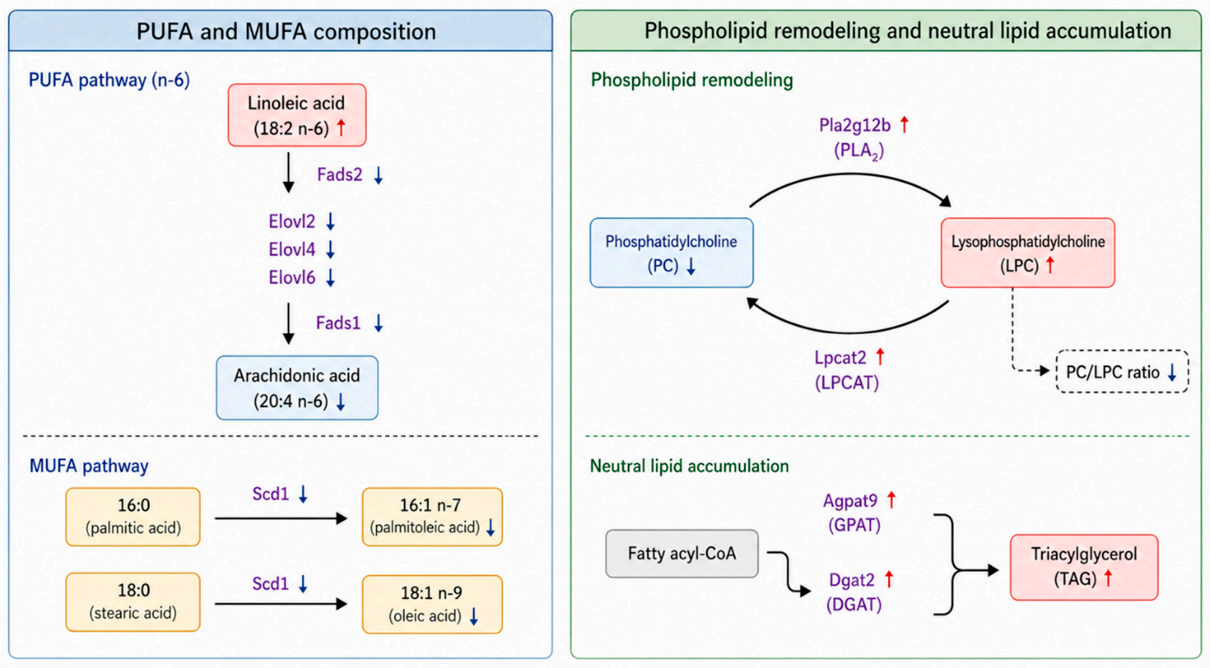

Figure S1. Schematic representation of integrated main lipidomic and transcriptomic changes in SR-B1 KO NTD embryos relative to WT embryos. ***Left panel:*** *PUFA and MUFA affected pathways.* ***Right panel:*** *pathways involving changes in phospholipid remodeling and neutral lipid accumulation. Symbols: ↑ increased; ↓ decreased in SR-B1 KO NTD vs WT. This figure was generated using ChatGPT (OpenAI) based on author-provided input and was reviewed and edited for scientific accuracy by the authors.*
